# Supplementary material for: Quantum Cutting in Ultraviolet B-Excited KY(CO3)2:Tb3+ Phosphors
Source: Materials (Basel). 2022 Sep 5;15(17):6160. doi: 10.3390/ma15176160 (PMC9457703; doi:10.3390/ma15176160)
Supplement: Supplementary file 1 [file materials-15-06160-s001.zip › materials-1878343-supplementary.pdf]

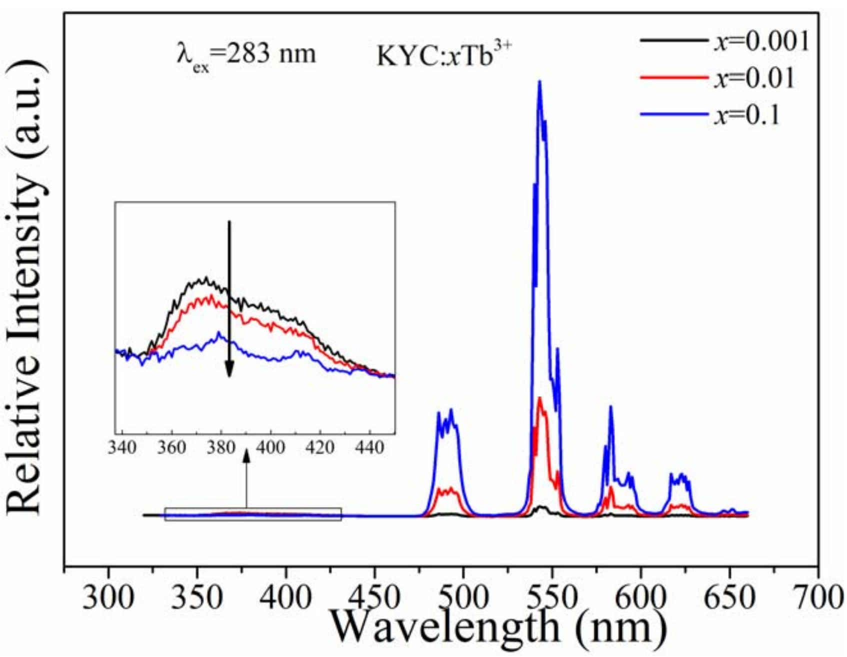

Figure S1. Emission spectra of KYC:xTb^{3+} (x=0.001, 0.01, 0.1) excited at 283 nm.

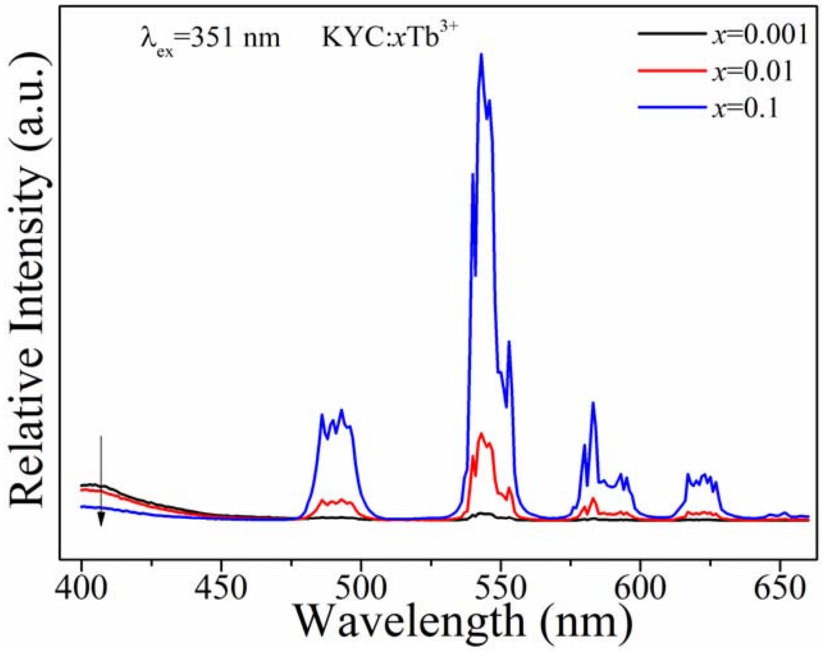

Figure S2. Emission spectra of KYC:xTb^{3+} (x=0.001, 0.01, 0.1) excited at 351 nm.
